# Genome-wide analysis of the *CBF* gene family and their transcriptional response to cold stress in *Hibiscus mutabilis*

**DOI:** 10.1038/s41598-025-05040-x

**Published:** 2025-07-03

**Authors:** Shiye Sang, Yuqiao Zhou, Yiqiong Liu

**Affiliations:** 1 Chengdu Botanical Garden (Chengdu Park Urban Institute of Plant Science), Chengdu, Sichuan China; 2 International Department of Chengdu Shude High School, Chengdu, Sichuan China

**Keywords:** *Hibiscus mutabilis*, CBF, Transcription factors, Low-temperature, Expression, Transcriptome, Computational biology and bioinformatics, Transcription

## Abstract

C-Repeat Binding Factors (*CBFs*) are crucial in plant responses to low-temperature stress via the ICE-CBF-COR cascade, but research on these genes in woody and flowering trees remains limited. *Hibiscus mutabilis*, a woody flowering plant of ornamental and ecological significance, faces low-temperature stress that substantially affects its growth and distribution. Understanding its cold tolerance mechanisms can enhance its utilization and provide insights into plant adaptability to climate change-induced agricultural challenges. This study presents the first genome-wide identification and characterization of the *CBF* gene family in *H. mutabilis*. Nine *HmCBF*s were identified, exhibiting uneven chromosomal distribution and clustering into five phylogenetic clades. *Cis*-regulatory element analysis indicated potential involvement of *HmCBFs* in abiotic stress responses and hormone signaling. Homology analysis indicated gene duplication during evolution and a close phylogenetic relationship between *H. mutabilis* and kenaf. Expression profiling demonstrated higher *HmCBF* expression in roots than in leaves under normal growth conditions, with significantly increased expression levels at 0 and − 5 °C compared to 5 °C following cold treatment. Our screening of *HmCBFs* in response to low-temperatures offers valuable insights for breeding cold-tolerant *H. mutabilis* and contributes to a broader understanding of stress tolerance mechanisms in woody species.

## Introduction

Low-temperatures are critical environmental stressors that adversely affect plant growth, development, geographic distribution, and overall productivity^1^. Cold stress, which predominantly occurs during early spring and winter, can substantially impair plant growth, reduce yields, and, in severe cases, result in plant mortality^2^. This stress is generally categorized into cold damage (> 0 °C) and freezing damage (< 0 °C)^3^. Both types of cold stress pose substantial threats to crop yield and quality by disrupting physiological processes. They suppress plant respiration and metabolism, reduce root water absorption, and cause symptoms such as leaf yellowing and fruit browning. Prolonged exposure to sub-zero temperatures (< 0 °C) damages cell membranes, leading to cell death. Even in the absence of freezing, temperatures above 0 °C can disturb water balance and physiological activities, ultimately causing plant injury or mortality^4^.

C-repeat binding factor (*CBF*) transcription factors, also known as DREB1 proteins, are members of the DREB subfamily within the AP2/ERF (APETALA2/ethylene-responsive factor) family. These transcription factors are rapidly inducible and play pivotal roles in plant responses to low-temperature stress. CBFs are characterized by a conserved AP2 domain and two unique signature sequences, PKKP(R)AGRxKFxETRHP and DSAWR^5^^6^,. Extensive studies have identified *CBF* genes across various plant species, including *Arabidopsis thaliana*^7^, *Capsicum annuum*^8^, *Oryza sativa*^9^, *Triticum aestivum*^10^, *Glycine max*^11^, *Vitis vinifera*^12^, *Camellia sinensis*^13^, and *Gossypium hirsutum*^14^. In *A. thaliana*, the CBF family comprises six members, among which AtCBF1, AtCBF2, and AtCBF3 are induced by low-temperatures and play crucial roles in enhancing freezing tolerance^7^. Conversely, AtCBF4, AtDDF2, and AtDDF1 are primarily activated by osmotic stresses, such as drought and salinity^15,16^.

The ICE-CBF-COR signaling cascade is a key pathway in CBF-mediated low-temperature responses^17^. Upstream transcription factor *ICE* induces *CBF* expression, which binds to the CRT/DRE (C-repeat/dehydration-responsive motif) *cis*-element in *COR* gene promoters, activating the expression of the downstream *COR* gene^18^. Functional studies have demonstrated that overexpression of AtCBF1 in *A. thaliana*^19^ and its sole introduction into tobacco (*Nicotiana tabacum*)^20^ and potato (*Solanum tuberosum*)^21^ substantially enhance cold tolerance in transgenic plants. Similarly, transgenic tobacco expressing GbCBF1 from cotton exhibits remarkably higher levels of free proline and soluble sugars under cold stress, substantially improving its cold tolerance^14^. Recent advancements in CBF research have revealed their critical role in mediating plant responses to cold stress within a complex signaling network including calcium signaling, epigenetic modifications, and hormonal interactions. Intracellular calcium influx, triggered by low-temperature conditions, has been shown to enhance CBF expression through calcium-responsive proteins, thereby enhancing the plant’s ability to withstand cold stress^4^. Epigenetic regulation also contributes substantially to cold-responsive gene activation. Histone modifications, particularly changes in H3K27me3 and H3K4me3 marks, influence the transcriptional activation of cold-responsive genes^22^. Additionally, the ICE1 protein plays a dual role in stress response by regulating CBF expression and interacting with components of the salicylic acid signaling pathway. This interaction not only strengthens abiotic stress tolerance but also enhances immunity against pathogens under cold stress conditions, underscoring the multifaceted role of CBFs in plant stress responses^23^. In tea plants (*C. sinensis*), the circadian rhythm regulator LUX ARRHYTHMO has been identified as a regulator of cold tolerance through its interaction with CBFs. This finding highlights the integration of circadian rhythms with cold stress responses, aligning physiological processes with environmental cues to optimize stress adaptation^4^.

*Hibiscus mutabilis*, a prominent member of the Malvaceae family, which includes species such as *Gossypium raimondii* and *Hibiscus syriacus* (Rose of Sharon), holds considerable cultural and ecological significance. Recognized as the city flower of Chengdu, *H. mutabilis* is highly valued for its ornamental traits, including its distinctive three-a-day color change, extended bloom duration, and unique floral morphogenesis. Beyond its aesthetic appeal, *H. mutabilis* has long been incorporated into traditional herbal remedies, attributed with properties that cool the blood, detoxify, reduce swelling, and alleviate pain. It has been traditionally employed in treating various ailments, such as ulcers, swelling, herpes zoster, scalds, and bruises^24^. Ecological surveys indicate that *H. mutabilis* predominantly thrives in south of the Yangtze River^25^, suggesting that its growth, development, and geographic distribution may be strongly influenced by environmental factors such as low-temperature stress. Cold stress is likely a critical determinant of the species’ growth, development, and geographic range. While the reference genome of *H. mutabilis* has been published, studies focusing on the expression and regulatory mechanisms of its CBFs remain unexplored^25^. Therefore, the present study aims to fill this knowledge gap by employing bioinformatics tools to systematically identify the *CBF* gene family in *H. mutabilis*. Further, it investigates the expression patterns of HmCBFs across various tissues under low-temperature stress through transcriptome sequencing and qRT-PCR analyses. These findings will provide a foundational basis for the cloning of HmCBFs and contribute to strategies aimed at enhancing the cold tolerance of *H. mutabil*is, with the goal of promoting the cultivation and application of *H. mutabilis* in a wider range of climatic regions. Furthermore, as an important resource plant with both ornamental and medicinal values, *H. mutabilis* demonstrates significant socioeconomic benefits in urban landscaping, traditional Chinese medicine industry, and ecological restoration. Its cultivation and application across broader climatic regions will enable multidimensional, multilevel, and wide-ranging exploitation and utilization of this valuable resource.

## Results

### Identification, characterization, and chromosome location

Using the six AtCBF proteins from *A. thaliana* as query sequences, a whole-genome scan of *H. mutabilis* was performed to identify protein-coding genes containing the CBF-specific domain. Both BLASTP and HMM search methods were employed, and candidate sequences lacking the “PKKRAGRxKFxETRHP” and “FADSAW” sequence tags were excluded. A total of nine *CBF* genes were identified (Table 1, see Supplementary Data S1 online). Among the identified genes, HmCBF4 encoded the longest protein sequence, comprising 230 (aa) amino acids, whereas HmCBF6 encoded the shortest sequence with 207 aa. The molecular weights of the identified CBF proteins ranged from 22.86 kDa (HmCBF6) to 25.67 kDa (HmCBF4), while their isoelectric points (pI) ranged from 5.24 (HmCBF3) to 6.97 (HmCBF9), indicating that these proteins are generally acidic. The instability index ranged from 42.54 to 62.19, exceeding the threshold of 40, classifying all identified CBF proteins as unstable. Subcellular localization predictions revealed that all CBF proteins localized to the nucleus, consistent with their expected roles as transcription factors (Table 1).

**Table 1:** Identification of *CBF* genes.

Gene name	Gene ID	AA^a^	MW^b^(kDa)	pI^c^	II^d^	GRAVY ^e^	SL^f^
*HmCBF*1	evm.model.ctg469.34	219	24.34	5.74	59.18	−0.697	Nucleus
*HmCBF*2	evm.model.ctg477.6	219	24.54	6	55.25	−0.573	Nucleus
*HmCBF*3	evm.model.ctg838.61	228	25.28	5.24	50.73	−0.630	Nucleus
*HmCBF*4	evm.model.ctg930.305	230	25.67	5.56	56.73	−0.572	Nucleus
*HmCBF*6	evm.model.ctg1003.4	207	22.86	5.28	62.19	−0.694	Nucleus
*HmCBF*5	evm.model.ctg1033.56	225	24.93	5.61	51.06	−0.589	Nucleus
*HmCBF*7	evm.model.ctg1351.449	218	24.43	6	54.02	−0.632	Nucleus
*HmCBF*8	evm.model.ctg1570.140	221	24.54	5.56	55.11	−0.561	Nucleus
*HmCBF*9	evm.model.ctg2067.16	220	24.59	6.97	42.54	−0.789	Nucleus

^a^Number of amino acids, ^b^Molecular weight, ^c^Theoretical isoelectric point, ^d^Instability index, ^e^Grand average of hydropathicity, ^f^Subcellular localization.

**Fig. 1:**
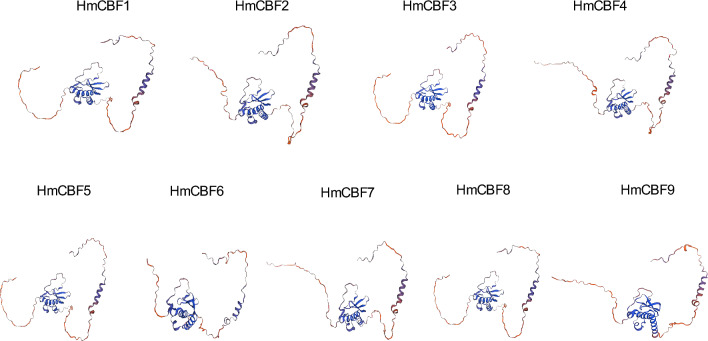
Tertiary structure models of *CBF* proteins of *H. mutabilis.* From left to right are the N-terminus and C-terminus. The classic AP2/ERF DNA-binding domain (with the typical β-sheet–α-helix–β-sheet topology) is shown in blue.

**Fig. 2:**
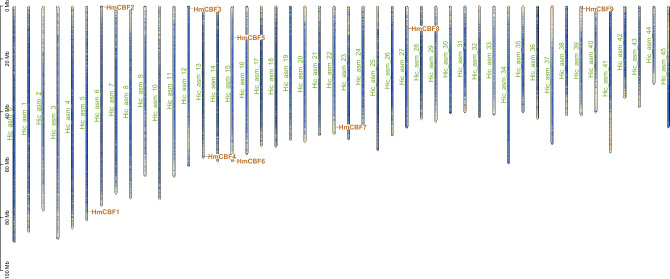
Chromosomal localiztion of *HmCBF* genes in the *H. mutabilis* genome. Hicasm 1–45 represents chromosome 1–45 of *H. mutabilis.*

The secondary structure prediction indicated that the *H. mutabilis* CBF proteins predominantly consisted of α-helices, β-sheets, random coils, and extended strands, arranged in the order of abundance: random coils > α-helices > extended strands > β-sheets (Table 2). The proportion of random coils ranged from 48.18% (HmCBF9) to 64.47% (HmCBF3), while that of α-helices ranged from 23.74% (HmCBF1) to 35.00% (HmCBF9). In comparison, the proportions of extended strands and β-sheets were lower, ranging from 10.09% (HmCBF3) to 16.00% (HmCBF5) and 0.00% (HmCBF3) to 5.00% (HmCBF9), respectively. Tertiary structure modeling analysis revealed a high degree of consistency among the nine HmCBF members (Fig. 1), suggesting no remarkable structural differences across the gene family. Chromosomal localization analysis showed that the *HmCBF* genes were unevenly distributed at two ends of eight chromosomes. Specifically, *HmCBF1*, *HmCBF2*, *HmCBF3*, *HmCBF4*, *HmCBF5*, *HmCBF6*, *HmCBF7*, *HmCBF8*, and *HmCBF9* were mapped to Chr5, Chr6, Chr12, Chr13, Chr15, Chr15, Chr22, Chr27, and Chr39, respectively (Fig. 2). The *CBF* family was not distributed across all 46 chromosomes, indicating an uneven chromosomal distribution. This unevenness highlights the complexity and potential diversification of the *CBF* gene family in *H. mutabilis*, providing valuable insights into their evolutionary processes.

**Table 2:** Secondary structure composition of *CBF* genes.

Gene	Alpha helix	Beta turn	Random coil	Extended strand
*HmCBF*1	23.74	3.65	56.62	15.98
*HmCBF*2	30.14	2.74	54.34	12.79
*HmCBF*3	25.44	0.00	64.47	10.09
*HmCBF*4	28.70	2.61	56.09	12.61
*HmCBF*6	30.43	4.35	53.62	11.59
*HmCBF*5	24.44	4.89	54.67	16.00
*HmCBF*7	23.85	3.21	58.72	14.22
*HmCBF*8	31.22	2.71	54.30	11.76
*HmCBF*9	35.00	5.00	48.18	11.82

### Phylogenetic analysis and classification

**Fig. 3:**
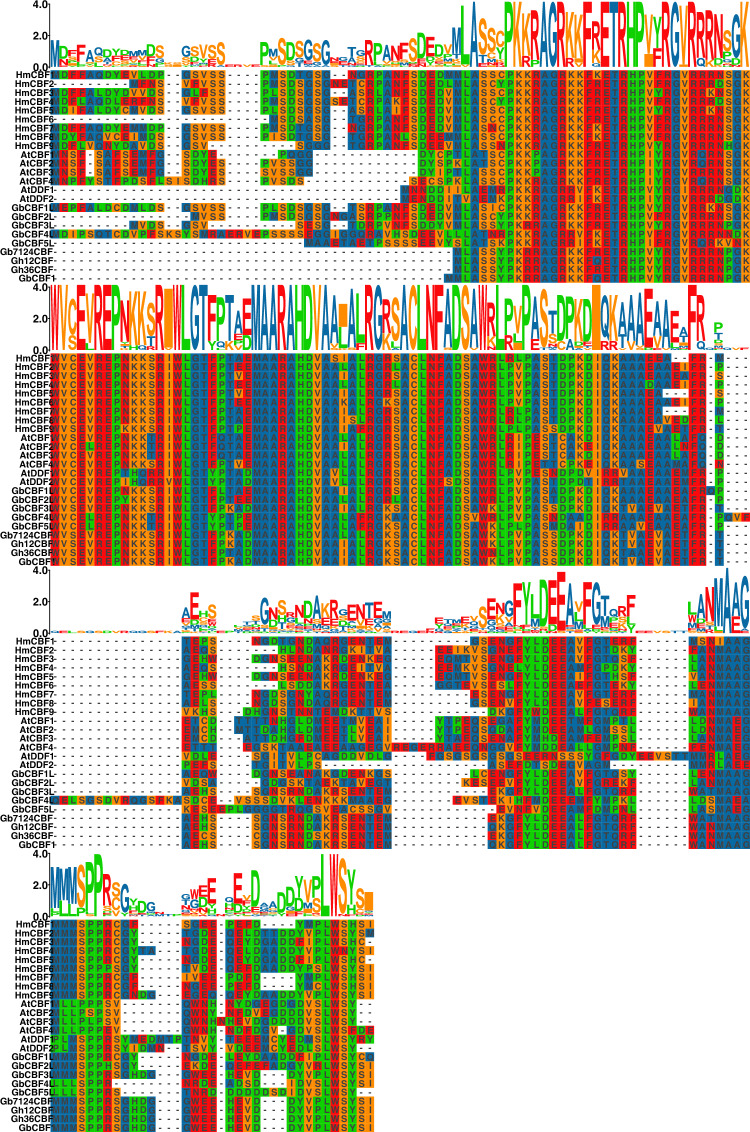
Multiple sequence alignment of CBF protein sequences.

**Fig. 4:**
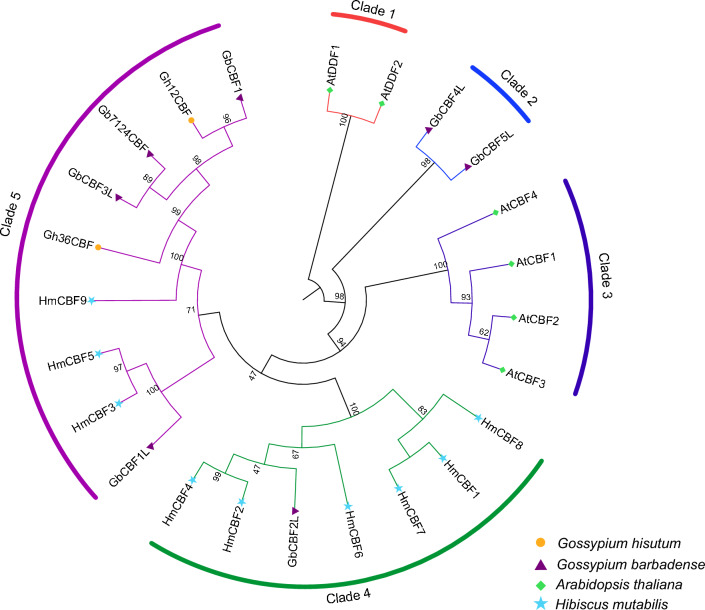
Phylogenetic tree of CBF proteins from four plant species—*Gossypium hirsutum*, *Gossypium barbadense*, *Arabidopsis thaliana*, and *H. mutabilis*. Different colors indicate distinct clades.

Phylogenetic analysis and classification of the *CBF* gene family were conducted to examine evolutionary relationships between *H. mutabilis* and other plant species (Figs. 3, 4). The phylogenetic tree was constructed using a total of 24 CBF proteins: nine from *H. mutabilis* (HmCBFs), nine from *G. hirsutum* and *G. barbadense* (GbCBFs and GhCBFs), and six from *A. thaliana* (AtCBFs and AtDDFs). These proteins were clustered into five distinct clades: clade 1–5. The *H. mutabilis* CBFs were exclusively grouped in clade 4 and clade 5, indicating species-specific clustering. Clade 4 has the highest number, containing six *H. mutabilis* CBFs (HmCBF1, HmCBF2 HmCBF4, HmCBF6, HmCBF7, and HmCBF8). Clade 5, the largest group, included six out of the nine CBFs from *G. hirsutum* and *G. barbadense* and three CBFs from *H. mutabilis* (HmCBF3, HmCBF5, and HmCBF9). Notably, all four *A. thaliana CBFs* genes were clustered within clade 3. AtDDF1 and AtDDF2 clustered in clade 1, while GbCBF4L and GbCBF5L grouped in clade 2. The CBFs from different species demonstrated close relationships within their respective clades, reflecting evolutionary conservation and potential functional similarities among these genes.

### Gene structure and conserved motif analysis

**Fig. 5:**
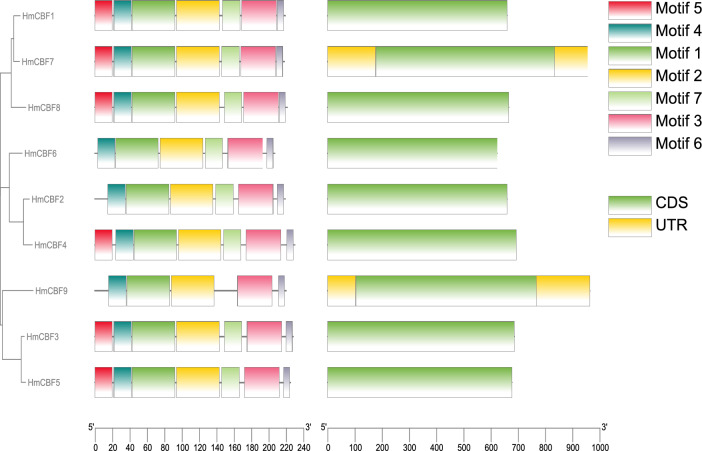
Gene structure and conserved motif analysis of *HmCBF* genes. The left panel shows a phylogenetic tree of the nine CBF proteins in *H. mutabilis*. The middle panel displays the motif composition of the *HmCBF* genes, while the right panel illustrates their gene structures.

Gene structure and conserved motif analyses were conducted to elucidate the architectural and functional organization of the *H. mutabilis CBF* gene family. The gene structures of the nine CBFs were ordered in alignment with the phylogenetic tree (Fig. 5, see Supplementary Fig. S1 online). All CBFs consisted of a single exon. The conserved motif analysis revealed the presence of seven motifs in six HmCBFs, whereas HmCBF2 and HmCBF6 contained six motifs, and HmCBF9 had only five motifs. Notably, all motifs, except Motif 5, were uniformly present across the CBFs. Motif 5 was absent in HmCBF2, HmCBF6, and HmCBF9, while Motif 7 was missing only in HmCBF9. These findings suggest a high degree of conservation in gene structure and motif composition among the *H. mutabilis* CBFs, with subtle variations potentially linked to specific functional diversities.

### Cis-acting elements of CBF genes

**Fig. 6:**
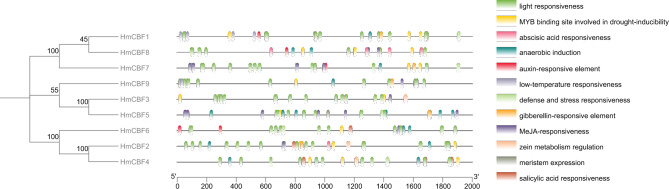
Distribution and frequency of *cis*-acting elements in the promoter regions of *HmCBF* genes. Different colored boxes represent distinct elements.

The analysis of *cis*-acting elements within the promoters of the nine *H. mutabilis CBF* genes identified 12 distinct types of regulatory elements (Fig. 6, see Supplementary Table S1 online). Low-temperature response elements were present in the promoters of two *HmCBFs* genes—*HmCBF1* and *HmCBF9*, highlighting their potential role in cold stress responses. Light-responsive elements were abundant in the promoters of all *CBF* genes, suggesting a widespread role in light-mediated regulation. Hormone-responsive elements were also prominently observed. Methyl jasmonate (MeJA)-responsive elements were found in the promoters of eight *CBFs*, excluding *HmCBF1*. Auxin-responsive elements were identified in the promoters of six *CBF* genes—*HmCBF1*, *HmCBF4*, *HmCBF5*, *HmCBF6*, *HmCBF7*, and *HmCBF8*, while abscisic acid-responsive elements were universally present in all *CBF* promoters. Gibberellin-responsive element was observed in five *CBFs* (*HmCBF1*, *HmCBF2*, *HmCBF5*, *HmCBF7*, and *HmCBF9*), and salicylic acid-responsive elements were detected in the promoters of *HmCBF2*, *HmCBF4*, and *HmCBF6*. For stress-related responses, defense and stress-responsive elements were identified in the promoters of five *CBF* genes (*HmCBF1*, *HmCBF4*, *HmCBF5*, *HmCBF6*, and *HmCBF7*), while drought-inducible elements were found in the promoters of all *CBF* genes. These findings indicate that *HmCBF* genes likely play significant roles in abiotic stress responses, including cold, drought, and light stress, as well as in hormonal signaling pathways associated with environmental adaptation.

### Homology analysis of CBF genes

**Fig. 7:**
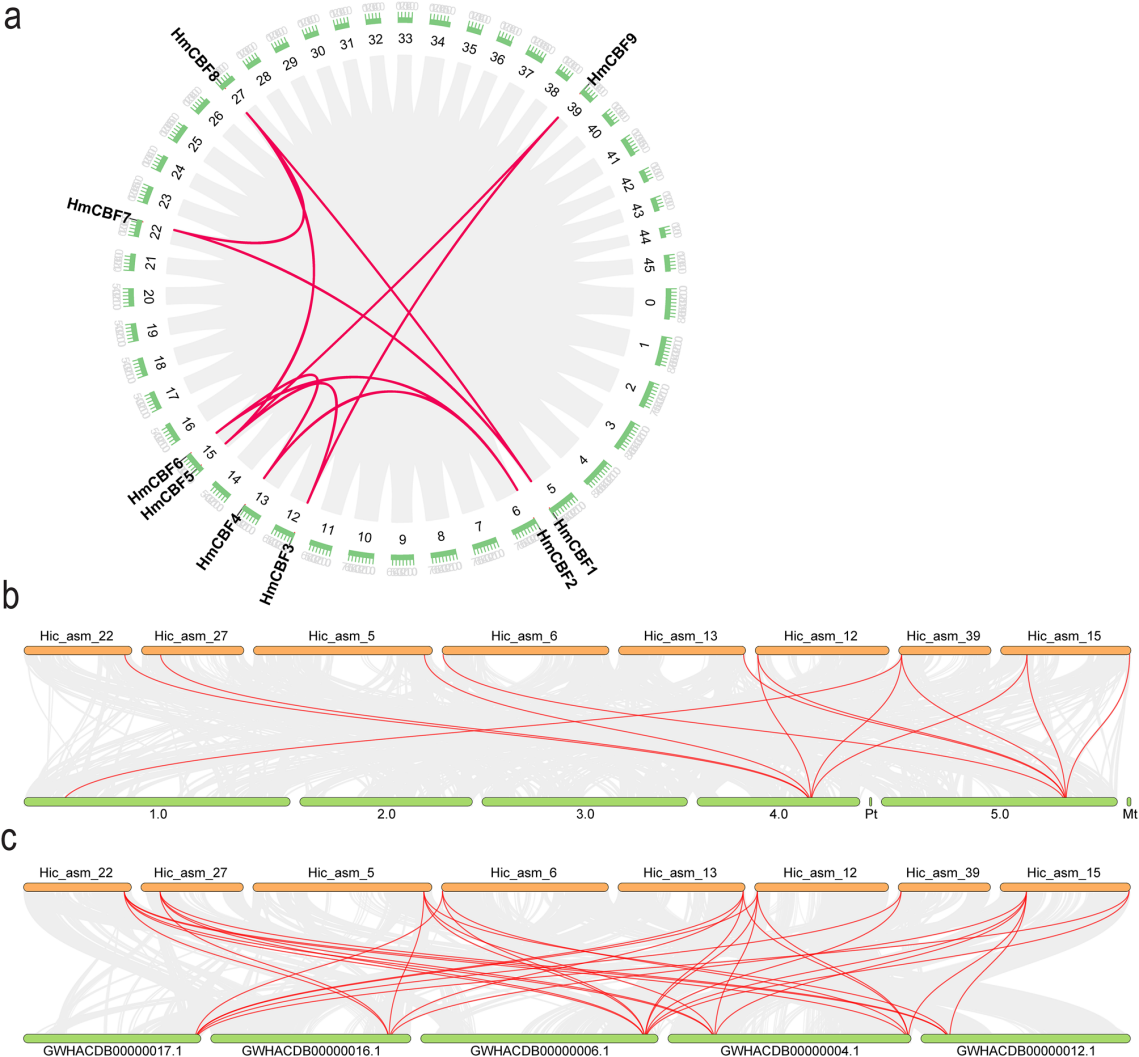
Synteny analysis of *CBF* genes in *H. mutabilis.* (**a**) Synteny among *CBF* genes within *H. mutabilis*. (**b**) Synteny between *H. mutabilis* and *A. thaliana*. (**c**) Synteny between *H. mutabilis* and kenaf (*H. cannabinus*). Red lines indicate homologous gene pairs.

The homology analysis of the *CBF* genes revealed both intraspecific and interspecific synteny relationships (Fig. 7, see Supplementary Table S2 online). Within *H. mutabilis*, nine *HmCBF* genes formed 10 homologous gene pairs, indicating a complex network of synteny (Fig. 7a). These pairs are likely the result of segmental duplications, classifying them as paralogous genes that have expanded during the evolutionary process. Interspecific synteny analysis further revealed evolutionary relationships. A comparison between *H. mutabilis* and *A. thaliana* identified synteny between all nine *HmCBF* genes and three *Arabidopsis* genes (*AtCBF2*, *AtCBF4*, and *AtDDF1*), resulting in 13 orthologous gene pairs (Fig. 7b). Moreover, reciprocal best hit (RBH) analysis confirmed that *AtCBF1* and *HmCBF7* are orthologous gene pairs, substantiating the reliability of the identified *HmCBF* genes. Similarly, synteny analysis between *H. mutabilis* and kenaf (*H. cannabinus*) demonstrated synteny between all nine *HmCBFs* and five *H. cannabinus* genes (GWHACDB00000006.1, GWHACDB00000004.1, GWHACDB00000012.1, GWHACDB00000016.1, and GWHACDB00000017.1), yielding 33 orthologous gene pairs (Fig. 7c). These findings suggest that *CBF* genes in *H. mutabilis* have undergone duplication events during evolution and suggest a close phylogenetic relationship between *H. mutabilis* and kenaf.

### Tissue-specific expression analysis of HmCBFs

**Fig. 8:**
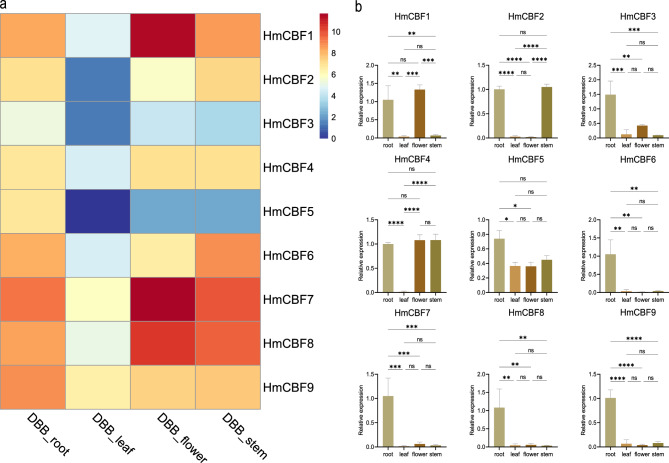
Expression profile of *HmCBF* genes in different tissues. (**a**) Transcriptome sequencing results, where blue, white, and red represent weak, medium, and strong gene expressions, respectively. (**b**) Gene expression levels validated by qRT-PCR. Error bars indicate standard errors. A single asterisk (*) denotes significant differences at the *P* < 0.05 level, with additional asterisks representing higher levels of significance. “ns” indicates no significant difference at the *P* < 0.05 level.

The tissue-specific expression analysis of *HmCBFs* was conducted using transcriptome sequencing and qRT-PCR to assess their expression profiles across different tissues of *H. mutabilis*. The qRT-PCR results for *HmCBF2*, *HmCBF3*, *HmCBF4*, *HmCBF5*, and *HmCBF9* were entirely consistent with the transcriptome sequencing data (Fig. 8, see Supplementary Table S3 online). The expression of *HmCBF2* was significantly downregulated in leaves and flowers compared to that in control (roots), while its expression in stems was comparable to that in roots. Similarly, *HmCBF4* exhibited downregulation in leaves but was highly expressed in flowers and stems, mirroring its expression in roots. In contrast, *HmCBF3*, *HmCBF5*, and *HmCBF9* showed downregulation in leaves, flowers, and stems compared to roots. For the remaining *HmCBFs* (*HmCBF1*, *HmCBF6*, *HmCBF7*, and *HmCBF8*), qRT-PCR results were partially consistent with sequencing data. Specifically, *HmCBF6* was downregulated in both leaves and flowers, while *HmCBF7* and *HmCBF8* displayed reduced expression in leaves. Overall, the expression levels of *HmCBFs* are higher in the roots and lower in the leaves.

### Expression of CBF genes under low-temperature conditions

**Fig. 9:**
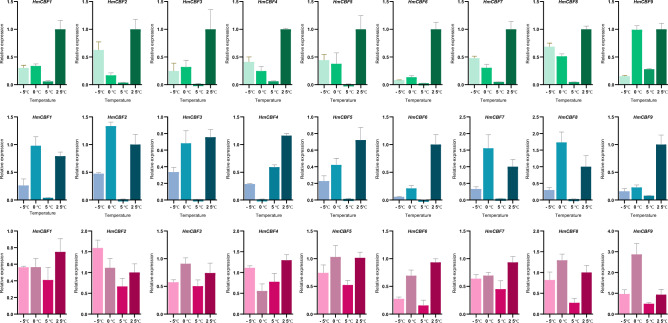
Expression profiles of *HmCBF* genes in various tissues under low-temperature stress. Green, blue, and red lines represent gene expression levels in leaves, roots, and stems, respectively. The Y-axis represents gene expression levels detected via qRT-PCR, while the X-axis indicates treatment temperatures. Error bars indicate standard errors. A single asterisk (*) denotes significant differences at the *P* < 0.05 level, with additional asterisks representing higher levels of significance. “ns” indicates no significant difference at the *P* < 0.05 level.

The expression of *HmCBF* genes under low-temperature stress was assessed through real-time quantitative PCR analysis in root, stem, and leaf tissues. These analyses aimed to elucidate the roles of *HmCBF* genes in cold stress responses, given their established importance in such conditions^26,27^. In leaves, all *HmCBF* genes, except *HmCBF9* at 0 °C, were significantly downregulated after 24 h of exposure to − 5 °C, 0 °C, and 5 °C compared to the control (25 °C), indicating that *HmCBF* genes indeed respond to low-temperature stress (Fig. 9, Supplementary File 5). Among these, *HmCBF2*, *HmCBF4*, *HmCBF5*, *HmCBF7* and *HmCBF8* exhibited higher expression levels with the severity of cold stress. In contrast, *HmCBF1*, *HmCBF6*, and *HmCBF9* exhibited peak expression levels at 0 °C among all low-temperature treatments. In roots, low-temperature stress generally led to the downregulation of *HmCBF* genes. Notable exceptions were *HmCBF2* and *HmCBF8*, which displayed upregulated expression under 0 °C stress, while *HmCBF1*, *HmCBF3*, *HmCBF5*, and *HmCBF7* showed no significant differences in expression under the same conditions. In stems, the expression profiles differed significantly from those in leaves and roots. Under all three cold stress conditions, *HmCBF* expression levels in stems relative to the control were higher than those in leaves and roots. Unlike in leaves, most *HmCBF* genes in stems did not show significant downregulation under low-temperature conditions. Remarkably, *HmCBF9* exhibited significantly higher expression at 0 °C compared to the control. When examining the overall expression profiles across roots, stems, and leaves, it was evident that *HmCBF* expression levels at 0 and − 5 °C were significantly higher than at 5 °C after 24 h of treatment. These findings highlight distinct expression patterns of *HmCBF* genes across tissues and stress levels, indicating their significant role in the cold stress response of *H. mutabilis*.

## Discussion

*Hibiscus mutabilis* is a valuable ornamental flower and medicinal herb, deeply embedded in the cultural heritage of Chengdu. However, its natural distribution is largely confined to regions south of the Yangtze River, implying that climatic factors, particularly temperature, may significantly limit its geographic range. Enhancing the adaptability of *H. mutabilis* to diverse environmental conditions is therefore crucial for expanding its cultivation. One promising approach involves leveraging the functional roles of transcription factors and other novel stress-responsive genes to improve germplasm resilience. For instance, *LEA* genes have been shown to enhance drought tolerance in cotton^28^. Similarly, *CBFs* are well-established as key regulators of plant responses to abiotic stresses, including salinity, drought, and low-temperatures. Among these, *CBFs* are particularly notable for conferring cold stress tolerance, as demonstrated in tree species, such as *Eucalyptus gunnii*, *Malus* × *domestica*, and *Betula pendula*^29,30,31^. Currently, we have established conditions for validating gene functions in *H. mutabilis*. Using a TRV-mediated VIGS system, we successfully silenced the *CLA1* gene associated with chloroplast development^32^. Therefore, the genome-wide identification and characterization of *CBFs* in *H. mutabilis* provide a pivotal step towards understanding their role in cold resistance mechanisms. This knowledge can be utilized to develop novel germplasm with enhanced cold tolerance, offering new opportunities for expanding the cultivation and utilization of *H. mutabilis*.

Although abiotic stress poses a significant challenge to the growth and survival of *H. mutabilis*, no studies have previously reported on its *CBF* genes. In contrast, the CBF family has been identified and studied in various plant species, including cotton^33^, wheat^34^, lettuce^35^, rapeseed^36^, barley^37^, and soybean^11^. In this study, a genome-wide identification, characterization, and expression analysis of the *H. mutabilis CBF* gene family was conducted, resulting in the identification of nine CBF proteins. Comparatively, the number of CBF proteins identified in *H. mutabilis* is less than those in plants such as lettuce (2.7 Gb genome, 14 CBF proteins), *Brassica napus* (1.2 Gb genome, 10 CBF proteins), soybean (1.1 Gb genome, 14 CBF proteins), and barley (5.1 Gb genome, 20 CBF proteins). However, it exceeds the numbers identified in *Medicago truncatula* (500 Mb genome, seven CBF proteins), *Phaseolus vulgaris* (587 Mb genome, seven CBF proteins), *Anacardium occidentalie* (671 Mb genome, six CBF proteins), and *Prunus sibirica* (230 Mb genome, four CBF proteins). This suggests a potential correlation between genome size and the number of *CBF* genes, as plants with larger genomes generally tend to encode more CBF proteins. Despite its relatively large genome size (2.68 Gb), *H. mutabilis* encodes fewer *CBF* genes than expected. This discrepancy may stem from the stringent screening criteria applied in this study, which prioritized precise identification of *CBF* genes. This highlights the importance of methodological rigor in accurately characterizing gene families across plant genomes. Further analysis revealed that the *H. mutabilis CBF* gene family shares conserved structural features with those of other dicotyledonous plants. Members of the *Arabidopsis CBF* family, for example, possess an AP2 domain flanked by conserved amino acid sequences upstream and downstream of the AP2 domain in each member. Upstream of the AP2 domain lies the PKK/PKKPAGR (RAGRxxKFxETRHP) motif, while the DSAWR motif is located downstream^5^^6^,. Mutations in the PKK/PKKPAGR motif have been shown to impair the binding of CBF proteins to the COR promoter of its downstream genes, thereby weakening their regulatory level function. These motifs are critical for CBF proteins to perform their role as transcription factors. The *CBF* gene family identified in *H. mutabilis* also exhibit conserved AP2/EREBP domains and characteristic CBF-like features, suggesting functional similarities with CBFs in other dicotyledonous plants.

It is widely recognized that transcription factors must localize to the nucleus to fulfill their functional roles^1^. Consistent with this, all *H. mutabilis* CBF proteins were localized in the nucleus (Table 2), aligning with their biological function as transcription factors. Analysis of gene structure and conserved motifs revealed that genes clustering on the same phylogenetic branch exhibited significant similarity in exon number, motif count, and motif position (Fig. 5). This observation suggests that conserved motifs play critical functional and/or structural roles in active proteins^38^. Notably, all *H. mutabilis CBF* family members were identified as intron-deleted genes. The absence of introns could potentially reduce the time required for transcription and translation, thereby accelerating protein production and enabling adaptation to environmental changes. Phylogenetic analysis classified the *H. mutabilis* CBF family into five distinct groups. All cotton CBFs included in the phylogenetic tree are known to respond to various abiotic stresses (including cold, drought, and high salinity). Specifically, GbCBF1L, GbCBF4L, and Gb7124CBF have been confirmed to be induced by low-temperature stress in island cotton^14,39^. Based on this clustering pattern, it is reasonable to speculate that HmCBF3, HmCBF5, and HmCBF9, which cluster with these cotton CBFs, may also be induced by cold stress. However, AtCBF1, AtCBF2, and AtCBF3, the *Arabidopsis* CBFs that are well-established as key regulators in cold-stress responses, did not cluster with any of the HmCBFs on the same branch. This divergence is likely attributable to the distant phylogenetic relationship between *Arabidopsis* and *H. mutabilis*. Nevertheless, a gene homology analysis revealed synteny between HmCBF1, HmCBF5, HmCBF7, HmCBF8, and HmCBF9 and AtCBF1, AtCBF2, and AtCBF3. These findings suggest that these HmCBFs are also likely to be involved in cold stress responses, highlighting the potential conservation of their functional roles across species despite phylogenetic divergence.

Gene promoters, located upstream of transcriptional start sites, contain numerous *cis*-acting elements that regulate gene transcription^40^. Promoter polymorphisms in *CBFs* significantly influence their expression, subsequently affecting the expression of downstream stress-responsive genes in *A. thaliana*^41,41,43^. For instance, studies have demonstrated that *AtCBF1*, *AtCBF2*, and *AtCBF3* are induced by low-temperatures, significantly enhancing the freezing tolerance of *A. thaliana*^7^. Meanwhile, *AtCBF4*, *AtDDF2*, and *AtDDF1* are primarily expressed in response to osmotic stress, such as drought and salinity^15,16^. In this study, we identified various *cis*-acting elements in the promoter regions of *HmCBF* genes, including those responsive to low-temperature, drought, light, and hormones. Notably, low-temperature responsive elements were found in the promoters of HmCBF1 and *HmCBF9*, suggesting that these genes might be significantly induced by cold stress. Additionally, we identified drought-inducible elements, defense and stress- responsive elements, and hormone-responsive elements (Fig. 6), which likely play a role in activating *CBFs* under various abiotic stresses. While we explored the expression patterns of *HmCBFs* under cold stress, we observed that most *HmCBFs* in both leaves and roots were downregulated following exposure to low temperatures for 24 h. This pattern aligns with findings of Long et al. regarding *CBF* gene expression levels in common beans and is partially consistent with Zhao et al. findings in three Acer species^44,45^. We hypothesize that this may reflect the rapid response of *HmCBFs* to cold stress, wherein their expression peaks shortly after exposure but declines over prolonged stress periods. This phenomenon has been reported in several studies, emphasizing that the initial induction of *CBF* genes during cold stress is often followed by downregulation as stress persists. However, a noteworthy observation was that the expression levels of *H. mutabilis* were significantly higher at 0 and − 5 °C compared to 5 °C, indicating that the stress response of *HmCBFs* is more pronounced at subzero temperatures. Moreover, expression levels of *HmCBFs* were consistently higher in roots of field-grown seedlings and lower in the leaves. This disparity might be attributed to the critical role of root systems in plant responses to environmental stress, including cold. Roots are central to regulating water and nutrient absorption, calcium signaling, and hormonal fluctuations, all of which contribute to temperature adaptation^46–49^.

Through the current study, while we have advanced our understanding of the *CBF* gene family’s expression profiles, the function of *HmCBFs* has not been further experimentally validated, and the underlying mechanisms driving these changes remain unclear. The function of *HmCBFs*, as well as the transcriptional dynamics of other genes within the ICE-CBF-COR cascade and the interactions among these genes, warrant further investigation. To address this, we are simultaneously conducting association analyses of genes in the ICE-CBF-COR cascade in *H. mutabilis* and performing targeted screening for candidate genes valuable for experimental validation. This integrated strategy will provide a more complete framework for understanding abiotic stress adaptation mechanisms in *H. mutabilis*.

## Materials & methods

### Plant materials and growth stress treatment

Two types of *H. mutabilis* cultivar ‘single-petal white’ samples were collected from the Chengdu Botanical Garden. The first group comprised field-grown seedlings that had naturally developed to the flowering stage, and roots, stems, leaves, and flowers were harvested for transcriptome sequencing, with roots serving as the control. The second group consisted of in vitro-grown seedlings propagated from seeds and cultivated under controlled greenhouse conditions for low-temperature stress treatment. The specific cultivation conditions for in vitro-grown seedlings are as follows: seeds of *H. mutabilis* were disinfected by immersion in 70% ethanol for 30 s, followed by rinsing with sterile water 3–4 times. They were then soaked in a 5.5% NaClO solution for 6–7 min and rinsed with sterile water 4–5 times to remove residual NaClO. Surface moisture was carefully blotted off before inoculating the seeds onto Murashige and Skoog (MS) culture medium. Seedlings were cultivated in a constant temperature incubator at 24–26 ℃ with a photoperiod of 14 h light and 10 h dark. After 60 d of growth, the seedlings were transferred to a climate chamber for low-temperature stress treatment. Experimental groups were established based on the temperature thresholds for cold damage (> 0 °C) and freezing damage (< 0 °C), as well as the typical temperature ranges in regions south of the Yangtze River (2 to 12 °C) and north of it (− 15 to 0 °C). Hence, the treatment temperatures were set at 5 °C, 0 °C, and − 5 °C, each maintained for 24 h under a photoperiod of 14 h light and 10 h darkness. The control group was maintained at 25 ℃ under identical light conditions for 24 h. Young leaves, roots, and stems were collected from each treatment group, flash-frozen in liquid nitrogen, and stored at − 80 ℃ for RNA extraction. Each treatment was performed with three biological replicates.

### Identification and characterization

To conduct a genome-wide analysis of *CBF* genes in *H. mutabilis*, the whole genome sequences were retrieved from public databases based on previously reported studies^25^. The CBF protein sequences were obtained from the *Arabidopsis* Information Resource (TAIR) database (https://www.arabidopsis.org/browse/genefamily/index.jsp)^50^. Both Hidden Markov Model (HMM) profiling^51^ and Basic Local Alignment Search Tool for Proteins (BLASTP)^52^ searches were conducted to identify *CBF* genes in *H. mutabilis*. The CBF-specific HMM profile (PF00847) was used with the HMMER toolkit (version 3.3.2, available at http://eddylab.org/software/hmmer) under default parameters, and BLASTP (version 2.14.1, available at https://ftp.ncbi.nlm.nih.gov/blast/executables/blast +) searches were performed using the AP2 domain sequences of six known *A. thaliana* CBF proteins as queries (e-value ≤ 1e-10). Conserved domains in the retrieved sequences were confirmed using the Simple Modular Architecture Research Tool (SMART) (Online tool, accessed in 2024; available at http://smart.embl-heidelberg.de/)^53^. Sequences containing the characteristic CBF-specific motifs— PKKRAGRxKFxETRHP and FADSAW—were retained for subsequent analysis.

The physicochemical properties of the identified CBF proteins, including protein length, molecular weight (kDa), and isoelectric point (pI), were predicted using the ExPASy Proteomics Server (ExPASy) (Online tool, accessed in 2024; available at https://prosite.expasy.org/)^54^. Secondary structure predictions were performed using the Self-Optimized Prediction Method With Alignment (SOPMA) (Online tool, accessed in 2024; available at https://npsa-prabi.ibcp.fr/cgi-bin/npsa_automat.pl?page=npsa_sopma.html), while tertiary structures were modeled using the SWISS-MODEL Workspace (SWISS-MODEL) (Online tool, accessed in 2024; available at https://swissmodel.expasy.org/interactive). Subcellular localization predictions were carried out using the WoLF (Web-based organism Localization Facility) PSORT (WoLF PSORT) (Online tool, accessed in 2024; available at https://wolfpsort.hgc.jp/).

### Phylogenetic and homology analysis

CBF protein sequences of *G. hirsutum* and *G. barbadense* were obtained from the Cottongen database (available at https://www.cottongen.org/)^55^, while the *Arabidopsis* CBF sequences were downloaded from NCBI (see Supplementary Table S4 online). Multiple protein sequences were aligned using Multiple Alignment using Fast Fourier Transform (MAFFT, version 7.490; available at https://mafft.cbrc.jp/alignment/software/)^56^ with default parameters, and subsequently trimmed using trimAI version1.4 (available at http://trimal.cgenomics.org/_media/)^57^. Phylogenetic trees were generated based on the alignment using the Maximum likelihood (ML) analysis using Intermediate Quantile (IQ)-TREE(IQ-TREE, version2.3.6; available at https://github.com/iqtree/iqtree2/releases/download)^58^, and the best-fit model was using ModelFinder for Phylogenetic Analysis (ModelFinder)^59^,with the JTT + G4 model and a bootstrap value of 1,000^60^. Collinearity analysis was conducted using the Multiple Collinearity Scan Toolkit software (McScanX, version: Integrated in TBtools [2017 version, equivalent to MCScanX official release]; available at https://github.com/wyp1125/MCScanX) with default parameters to identify syntenic relationships among the genomes. The resulting data were visualized using the Toolkit for Biologists (TBtools, version 2.136; available at https://github.com/CJ-Chen/TBtools/releases/latest ), enabling a comprehensive interpretation of genome-wide collinear regions. Orthologous gene pairs between AtCBFs and HmCBFs were identified through RBH analysis using BLASTP. A relatively non-stringent E-value threshold of 1e-10 was employed to capture potential homologous relationships^61^ .

### Conserved motifs and gene structure analysis

The conserved motifs of candidate CBF proteins were identified using the Multiple Expectation Maximization for Motif Elucidation tool (MEME, Online tool, accessed in 2025; available at http://meme-suite.org/tools/meme)^62^. The analysis parameters were configured as follows: minimum width of six, maximum width of 50, motif number set to seven, and a minimum of two sites per motif. The motif composition of the candidate CBFs was visualized using TBtools version 2.136, facilitating a comparative analysis of conserved regions^63^.

### Chromosomal localization and cis-acting elements

The chromosome positions of *CBF* genes in *H. mutabilis* were determined based on their physical positions (bp) within the genome. Promoter sequences, defined as the 2000 bp regions upstream of the coding sequences of *CBF* genes, were extracted using TBtools version2.136. The extracted promoter sequences were analyzed for *cis*-acting elements using the Plant Cis-Acting Regulatory Element Database (Plant CARE, Online tool, accessed in 2024; available at http://bioinformatics.psb.ugent.be/webtools/plantcare/html). Identified *cis*-elements were subsequently visualized using TBtools version2.136 to highlight the regulatory regions associated with *CBF* genes.

### Transcriptome sequencing

Samples were collected from four tissues—roots, stems, leaves, and flowers—of field-grown *H. mutabilis* seedlings. These samples were immediately frozen in liquid nitrogen to preserve RNA integrity for subsequent sequencing and RT-qPCR. Total RNA was extracted using the RNAprep Pure Plant Plus Kit (Polysaccharides & Polyphenolics-rich) (TIANGEN Biotech, Beijing, China) following the manufacturer’s protocol. The quality and quantity of the extracted RNA were evaluated using NanoPhotometer (Implen, Westlake Village, CA, USA), a Qubit 3.0 Fluorometer (Thermo Fisher Scientific, Carlsbad, CA, USA), and an Agilent 2100 Bioanalyzer (Agilent Technologies, Santa Clara, CA, USA). Only RNA samples with integrity values (RIN) greater than 7.0 were selected for subsequent cDNA library construction and sequencing. The cDNA libraries were constructed using the NEBNext Ultra RNA Library Prep Kit (E7350L, NEB, Ipswich, MA, USA) according to the manufacturer’s guidelines. Sequencing was performed on the Illumina NovaSeq 6000 platform (Illumina, San Diego, CA, USA) to generate high-quality 150-bp paired-end reads. The sequencing data achieved a clean Q30 base rate of 94.54%, with a mapping rate of 87.18%.

### Quantitative real-time PCR

Specific primers for the *HmCBFs* and internal reference gene *Actin* were designed based on the coding sequence (CDS) sequences of the *CBF* gene family in *H. mutabilis*^32^. Primer design was performed using Primer 6.0, and the primers were synthesized by Sangon Biotech Co., Ltd. (Shanghai, China) (Table 3). The *Actin* gene sequence was derived from *H. cannabinus* cultivar P3A actin 3 (*ACT3*) mRNA (Sequence ID: KX689256.1), and the corresponding *H. mutabilis* sequence (cds.evm.model.ctg200.70) was identified through BLAST. Total RNA was extracted from all the collected samples using the FastPure Plant Total RNA Isolation Kit (Polysaccharides & Polyphenolics-rich) (Vazyme, Nanjing, China). First-strand cDNA was synthesized using the HiScript II 1st Strand cDNA Synthesis Kit (Vazyme). The synthesized cDNA was diluted and stored for subsequent analysis. qPCR analysis was performed using the Taq Pro Universal SYBR qPCR Master Mix (Vazyme) in the QuantStudio1 real-time qPCR system (Thermo Fisher Scientific). The 20 μL PCR reaction mixture consisted of 2 μL cDNA, 0.4 μL each of 10 μM upstream and downstream primers, 10 μL of 2 × Taq Pro Universal SYBR qPCR Master Mix, and 7.2 μL of ddH2O. The amplification procedure for qRT-PCR included an initial pre-denaturation at 95 °C for 30 s, followed by 40 cycles of denaturation at 95 °C for 10 s and annealing at 60 °C for 30 s, with each sample analyzed in triplicate. The relative expression levels of each gene were calculated using the 2-ΔΔCt method. Expression plots were generated using GraphPad Prism: Scientific 2D Graphing and Statistics Software(GraphPad Prism, version 9.5; available at https://www.graphpad.com/download) to visualize the relative expression levels of *HmCBFs* under different experimental conditions.

**Table 3:** qRT-PCR primers for *H. mutabilis CBF* gene family.

*Gene name*	*Forward sequence*	*Reverse sequence*
*HmCBF1*	*CTGGTAGTGGAAATGGGCGT*	*CCCAGCCAAATCCTCGACTT*
*HmCBF2*	*GCGTTAGCCCTGAGAGGAAG*	*ACCTCTGTTTGCGTCGTTCA*
*HmCBF3*	*CTCCCAACCATAAACTCCAACT*	*GACTAGCTCCACTCCCGGAAT*
*HmCBF4*	*AATTCCGGGAGACACGACAC*	*CTTCCTCTCAGGGCTAACGC*
*HmCBF5*	*ATGACAGTGTCGGAGAATGGA*	*ATCGTCGGCACCGTCGTATTC*
*HmCBF6*	*CCCGCTGAGTCATCGCTTTCG*	*AAACTTTCCGACACTTCCGTTCCC*
*HmCBF7*	*GTGAAGTGAGGGAGCCTAACAA*	*TCGGCGAAGTTCAGACAAGC*
*HmCBF8*	*GGACGAGGAGATGATGTTGGCTTC*	*CCCTCACTTCACAAACCCACTTCC*
*HmCBF9*	*AAGTGGGTTTCCGAGGTGAG*	*GACAAGCCGACCTTCCTCTA*
*Actin*	*GCGATTCAGATGCCCAGAAGTCC*	*TGCCACCACTAAGCACGATGTTG*

## Conclusions

This study presents the first comprehensive identification and characterization of the *CBF* gene family in *H. mutabilis*, mapping the expression profiles of *HmCBFs* across various tissues and temperature conditions. A total of nine *CBF* genes were identified and analyzed in terms of their physicochemical properties, protein structure, subcellular localization, and chromosomal distribution. Further analyses, including phylogenetic, gene structure, conserved domain, *cis*-acting element, and qRT-PCR expression analyses, provided valuable insights into the potential biological functions of these genes. The findings from this study offer a foundational understanding of *CBF* genes in *H. mutabilis*, which is critical for advancing cold tolerance research. These results could facilitate the development of cold-tolerant germplasm, enhancing the species’ adaptability to low-temperature environments. Additionally, the outcomes hold potential for extending the ornamental period and expanding the geographical distribution of *H. mutabilis*, contributing to its broader cultivation and application.

## Supplementary Information

Supplementary Information 1.

Supplementary Information 2.

Supplementary Information 3.

Supplementary Information 4.

Supplementary Information 5.

Supplementary Information 6.

## Data Availability

Data is contained within the article or Supplementary Materials. The raw sequence data reported in this paper have been deposited in the Genome Sequence Archive^64^ in National Genomics Data Center, China National Center for Bioinformation / Beijing Institute of Genomics, Chinese Academy of Sciences (GSA: CRA024609) that are publicly accessible at https://ngdc.cncb.ac.cn/gsa.
