# Supplementary material for: Genome-wide analysis of the CBF gene family and their transcriptional response to cold stress in Hibiscus mutabilis
Source: Sci Rep. 2025 Jul 3;15:23808. doi: 10.1038/s41598-025-05040-x (PMC12229554; doi:10.1038/s41598-025-05040-x)
Supplement: Supplementary file 2 — Supplementary Information 2. [file 41598_2025_5040_MOESM2_ESM.pdf]

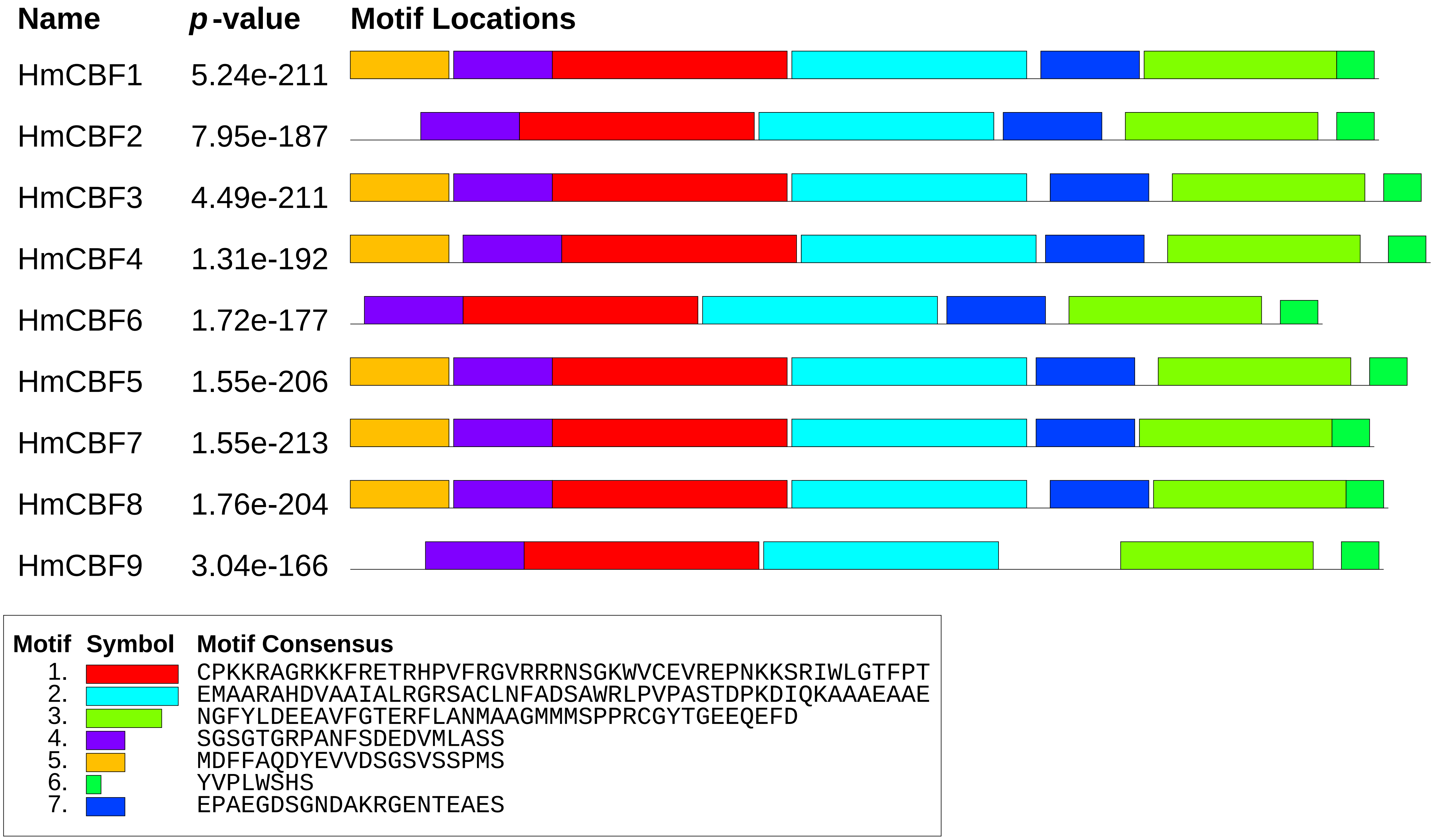
Name
p-value
Motif Locations
HmCBF1 5.24e-211
HmCBF2 7.95e-187
HmCBF3 4.49e-211
HmCBF4 1.31e-192
HmCBF6 1.72e-177
HmCBF5 1.55e-206
HmCBF7 1.55e-213
HmCBF8 1.76e-204
HmCBF9 3.04e-166
Motif Symbol Motif Consensus
1. CPKKRAGRKKFRETRHPVFRGVRRRNSGKWVCEVREPNKKSRIWLGTFPT
2. EMAARAHDVAAIALRGRSACLNFADSAWRLPVPASTDPKDIQKAAAEAAE
3. NGFYLDEEAVFGTERFLANMAAGMMMSPPRCGYTGEEQEFD
4. SGSGTGRPANFSDEDVMLASS
5. MDFFAQDYEVVDSGSVSSPMS
6. YVPLWSHS
7. EPAEGDSGNDAKRGENTEAES
